# Genome Evolution of Two Genetically Homogeneous Infectious Bursal Disease Virus Strains During Passages *in vitro* and *ex vivo* in the Presence of a Mutagenic Nucleoside Analog

**DOI:** 10.3389/fmicb.2021.678563

**Published:** 2021-06-11

**Authors:** Liliana L. Cubas-Gaona, Alexandre Flageul, Céline Courtillon, Francois-Xavier Briand, Maud Contrant, Stephanie Bougeard, Pierrick Lucas, Hélène Quenault, Aurélie Leroux, Alassane Keita, Michel Amelot, Béatrice Grasland, Yannick Blanchard, Nicolas Eterradossi, Paul Alun Brown, Sébastien Mathieu Soubies

**Affiliations:** ^1^Avian and Rabbit Virology, Immunology and Parasitology Unit (VIPAC), French Agency for Food, Environmental and Occupational Heath Safety (ANSES), Ploufragan, France; ^2^Viral Genetics and Biosecurity Unit (GVB), French Agency for Food, Environmental and Occupational Heath Safety (ANSES), Ploufragan, France; ^3^Epidemiology, Animal Health and Welfare Unit (EPISABE), French Agency for Food, Environmental and Occupational Heath Safety (ANSES), Ploufragan, France; ^4^Experimental Poultry Unit (SELEAC), French Agency for Food, Environmental and Occupational Heath Safety (ANSES), Ploufragan, France

**Keywords:** IBDV, chicken B cells, evolution, 7DMA, viral diversity, mutation frequency, passages

## Abstract

The *avibirnavirus* infectious bursal disease virus (IBDV) is responsible for a highly contagious and sometimes lethal disease of chickens (*Gallus gallus*). IBDV genetic variation is well-described for both field and live-attenuated vaccine strains, however, the dynamics and selection pressures behind this genetic evolution remain poorly documented. Here, genetically homogeneous virus stocks were generated using reverse genetics for a very virulent strain, rvv, and a vaccine-related strain, rCu-1. These viruses were serially passaged at controlled multiplicities of infection in several biological systems, including primary chickens B cells, the main cell type targeted by IBDV *in vivo*. Passages were also performed in the absence or presence of a strong selective pressure using the antiviral nucleoside analog 7-deaza-2′-C-methyladenosine (7DMA). Next Generation Sequencing (NGS) of viral genomes after the last passage in each biological system revealed that (i) a higher viral diversity was generated in segment A than in segment B, regardless 7DMA treatment and viral strain, (ii) diversity in segment B was increased by 7DMA treatment in both viruses, (iii) passaging of IBDV in primary chicken B cells, regardless of 7DMA treatment, did not select cell-culture adapted variants of rvv, preserving its capsid protein (VP2) properties, (iv) mutations in coding and non-coding regions of rCu-1 segment A could potentially associate to higher viral fitness, and (v) a specific selection, upon 7DMA addition, of a Thr329Ala substitution occurred in the viral polymerase VP1. The latter change, together with Ala270Thr change in VP2, proved to be associated with viral attenuation *in vivo*. These results identify genome sequences that are important for IBDV evolution in response to selection pressures. Such information will help tailor better strategies for controlling IBDV infection in chickens.

## Introduction

Infectious bursal disease (IBD) is caused by IBD virus (IBDV) and was observed for the first time in Delaware (United States) in 1957 (Cosgrove, 1962). This highly contagious disease affects young chickens, representing a serious threat to the poultry industry worldwide. After infection by the oral route, IBDV replicates in several lymphoid organs; the Bursa of Fabricius (BF) (a bird-specific primary lymphoid organ where the maturation of avian B cells occurs) is IBDV main target. Once in the BF, IBDV replicates actively in immature IgM^+^ B cells, causing bursal atrophy, immunosuppression and sometimes death (Dey et al., 2019).

Infectious bursal disease virus belongs to the family *Birnaviridae*, genus *Avibirnavirus*, and is a naked and bisegmented double-stranded RNA virus. Segment A (3.2 kbp) encodes two open reading frames (ORFs), which are partially overlapping. The first ORF encodes the 21 kDa non-structural viral protein 5 (VP5), while the second one codes for a 110 kDa polyprotein which is further cleaved by autoproteolysis to yield mature VP2 (capsid protein, 42 kDa), VP4 (protease, 28 kDa) and VP3 (scaffold protein, 32 kDa). The smaller segment B (2.8 kbp) encodes VP1 (97 kDa), the RNA-dependent RNA polymerase, RdRp (Dey et al., 2019; Eterradossi and Saif, 2020). VP2 contains a hypervariable region, hVP2, which carries the immunogenic determinants and spans from amino acid position 206 to 350 (Bayliss et al., 1990; Coulibaly et al., 2005; Dey et al., 2019). Mutations in hVP2 positions 253, 279, 284 and 330 have been related with both adaptation to avian and mammalian cell culture and viral attenuation in chickens of pathogenic field strains (Lim et al., 1999; van Loon et al., 2002; Noor et al., 2014).

Based on virus neutralization assays, IBDV strains are classified as belonging to serotype 1 or 2. Serotype 1 viruses can infect chickens, guinea fowls, ducks, quails, turkeys, pheasants and ostriches (Dey et al., 2019; Eterradossi and Saif, 2020). However, clinical forms of IBD, observed after infection by serotype 1 viruses, have only been reported in young chickens (3–8 weeks of age). Although infections by serotype 2 viruses have been demonstrated in chickens, ducks, fowls and turkeys (McFerran et al., 1980), these viruses are non-pathogenic. Depending on their pathogenicity in chickens, viruses from serotype 1 are further classified as classical virulent (cvIBDV), very virulent (vvIBDV) or subclinical (antigenic variants). IBD was effectively controlled until the 1980s by vaccines developed against classical strains. However, the emergence of vvIBDV in Europe (Chettle et al., 1989) and antigenic variants in the United States (Jackwood and Saif, 1987) during the 80s resulted in economic losses and high mortality in chickens. Since then, vvIBDV have spread and been reported in many parts of the world (Dey et al., 2019). Questions about unique genetic mutations involved in the evolution and expansion of vvIBDV remain open. Attenuated vaccines currently used to protect from IBD elicit a more robust immune response when compared to inactivated vaccines, but they convey the risk of reversion toward higher virulence (Olesen et al., 2018; Dey et al., 2019; Eterradossi and Saif, 2020). Thus, a better knowledge of the evolution of IBDV as well as new strategies in the development of safer and more stable vaccines are required to improve the control of emerging IBDV strains.

The relatively low intrinsic fidelity of RdRp is responsible in large part for the high mutation rates in RNA viruses, which appear to be the highest of all organisms (Borderia et al., 2016). These high mutation rates contribute to the generation of complex and dynamic mutant spectra, which facilitate virus adaptation and evolution in response to selection pressures. However, there is a maximum error rate, also called error threshold, above which genetic information can no longer be maintained, resulting in an error catastrophe and an abortive infection (Eigen, 2002; Domingo and Perales, 2019). Lethal mutagenesis is a particular case of error catastrophe. In this situation, during viral genome replication, mutagenic nucleoside analogs are incorporated by viral polymerases into the nascent strand of RNA, resulting in the accumulation of mutations above the error threshold, which is lethal for the virus. This concept explains the interest of nucleoside analogs as antiviral treatments (Crotty et al., 2000). However, the use of moderately toxic nucleoside analog concentrations can select resistant mutants. This resistant phenotype is often explained by mutations in the viral polymerase gene that alter the polymerase intrinsic fidelity, resulting in an expansion (low fidelity) or reduction (high fidelity) of the viral population diversity (Borderia et al., 2016). The study of those fidelity mutants has highlighted the role of mutant spectrum on the pathogenesis, adaptability to new environments and viral evolution of several RNA viruses. Fidelity mutants were for instance reported for poliovirus (Pfeiffer and Kirkegaard, 2003), Coxsackie virus B3 (Levi et al., 2010; Gnadig et al., 2012), foot-and-mouth disease virus (Zeng et al., 2013), chikungunya (Coffey and Vignuzzi, 2010), influenza A (Cheung et al., 2014) or West Nile (Van Slyke et al., 2015) viruses.

In the present study, two strains of IBDV produced from cloned DNA in a reverse genetics system (Cubas-Gaona et al., 2020) were used: one based on a non-passaged (p0), cell-culture adapted, attenuated, vaccine-related and classical strain (rCu-1-p0), and a second one based on a non-passaged, non-cell-culture adapted and very virulent strain (rvv-p0). rCu-1-p0 was serially passaged in DF-1 cells, chicken bursal cells (hereafter referred as chicken B cells), and chickens while rvv-p0 was exclusively passaged using chicken B cells to address four questions: (i) whether serial passaging of a homogeneous rvv-p0 virus population would select mutations previously observed upon wild-type heterogeneous vvIBDV adaptation to avian and mammalian cell cultures, (ii) whether serial passaging of homogeneous rCu-1-p0 virus population would select mutations associated to a reversion toward virulence of some heterogeneous live-attenuated vaccines, (iii) whether serial passaging in the presence of the adenosine analog 7-deaza-2′-C-methyladenosine (7DMA) would generate resistant mutants in one or both homogeneous virus populations, and (iv) whether potential resistant mutants would show an attenuated phenotype *in vivo*.

Viral stocks produced before and after serial passages were deep-sequenced to explore the composition of their viral populations and provide insights in the evolutionary trajectories, genetic variability and host adaptability of IBDV.

## Materials and Methods

### Cell and Viruses

The chicken fibroblast DF-1 cell line (ATCC CRL-12203) was grown in Dubelcco’s modified minimal essential medium (DMEM) (reference 61965-023, Thermo Fisher) supplemented with 10% fetal bovine serum (FBS), penicillin (200 IU/mL), streptomycin (0.2 mg/mL), fungizone (2 μg/mL) and maintained at 39 °C in a humidified 5% CO_2_ incubator.

The rescue of recombinant viruses rCu-1-p0 and rvv-p0 has been described recently (Cubas-Gaona et al., 2020). Briefly, DF-1 cells were transfected with recombinant expression vectors prACu-1 and prBCu-1 for rCu-1-p0 recovery or prAvv and prBvv for rvv-p0 recovery. After 72 h, cellular material was transferred onto B cells. At 48 h post transfer, the supernatants were recovered, aliquoted and titrated on B cells. The viral sequences used for rvv rescue are available in Genbank with the accession number MG489893 (for segment A) and MG489892 (for segment B). The viral sequences used for rCu-1 rescue are presented in Supplementary Table 1. The classical virulent strain rCu-1 presents more than 99% identity for both segments A and B with D78 and Cu-1 M, two live-attenuated vaccines used in Europe.

### Isolation of Primary Chickens Bursal Cells

All animal trials were conducted in an animal facility approved for animal experiments (n° C-22–745–1) and were approved by ANSES Ploufragan local committee for animal welfare; chickens were raised and humanely euthanized in agreement with EU directive number 2010/63/UE.

For experimental passages and viral titrations, BF were aseptically collected from four to ten week-old specific-pathogen-free (SPF) White Leghorns chickens (ANSES, Ploufragan, France) and were processed as previously described (Soubies et al., 2018). Bursal cells were maintained in lymphocyte culture medium at 40 °C in a humidified 5% CO_2_ incubator. This medium was prepared using Iscove’s modified Dulbecco’s Medium (IMDM) with L-glutamine and HEPES (reference 21980–032, Gibco, Thermo Fisher) supplemented with 8% FBS, 2% SPF chicken serum (ANSES, Ploufragan, France), 1X insulin transferrin selenium (reference 41400–045, Gibco, Thermo Fisher), 50 μM beta-mercaptoethanol, 1 μg/mL Phorbol 12-myristate 13-acetate (PMA, reference tlrl-pma, Invivogen), penicillin (200 IU/mL), streptomycin (0.2 mg/mL) and fungizone (2 μg/mL). PMA was reconstituted as previously described (Soubies et al., 2018). Bursal cells were diluted into phosphate-buffered saline (PBS) containing 0.1% (m/v) erythrosin B (reference 200964, Sigma-Aldrich) and counted in a Neubauer chamber to estimate cell viability and numbers after isolation.

### Virus Titration by Immunocytochemistry (ICC)

Ten-fold serial dilutions of viral stocks and of supernatants from cells in IMDM were distributed into 96-well U bottom plates (50 μL/well, eight replicates per viral sample). Freshly prepared chicken B cells in lymphocyte culture medium (10^6^ cells in 150 μL/well) were added in each well and incubated at 40 °C for 48 h in a humidified 5% CO_2_ incubator. Forty-eight hours post-infection (h.p.i.), the cells were washed with PBS and fixed with ethanol and acetone solution (1:1 ratio) at −20°C for at least 30 min. After removal of the fixation solution, the plates were air-dried under a chemical hood and processed immediately or stored at −20°C until further processing. The plates were subjected to ICC as previously described (Soubies et al., 2018). Viral titers expressed as TCID_50_/mL were determined using Reed and Muench formula (Reed and Muench, 1938).

### Experimental Passages in Cells and in Chickens

Either 5 × 10^6^ DF-1 or 5 × 10^7^ chicken B cells were seeded in 10 mL of their respective complete medium, as indicated above, on 25 cm^2^ flasks. Chicken B cells were promptly subjected to infection after isolation, while DF-1 cells were seeded 5 h before infection. DF-1 and chicken B cells were infected in 1 mL medium at a multiplicity of infection (MOI) of 0.1 and 0.01, respectively. After 1 h of viral adsorption in 1 mL medium with frequent rocking, the inoculum was removed. Fresh medium with or without 7-deaza-2′-C-methyladenosine (7DMA, reference ND08351, Carbosynth) at 2.5 μM for rCu-1 and 4 μM for rvv was added. The optimal non-toxic concentration of 7DMA was determined following the recommendations previously described (Beaucourt et al., 2011). After 24 h (chicken B cells) or 48 h (DF-1 cells), cells debris were removed by centrifugation and the supernatants were harvested and aliquoted before freezing at −70°C. One aliquot was titrated by ICC, as indicated above, to determine the required inoculum dilution for the next passage. rCu-1-p0 was passaged twelve times in parallel in chicken B cells and DF-1 cells (MOI of 0.01 and 0.1 at each passage, respectively). rvv-p0 was passaged twelve times at a constant MOI of 0.01 in chicken B cells only, since this strain does not replicate in DF-1 cells. Three parallel and independent stocks (called R1, R2 and R3, respectively) were produced for each condition and passage.

**FIGURE 1:**
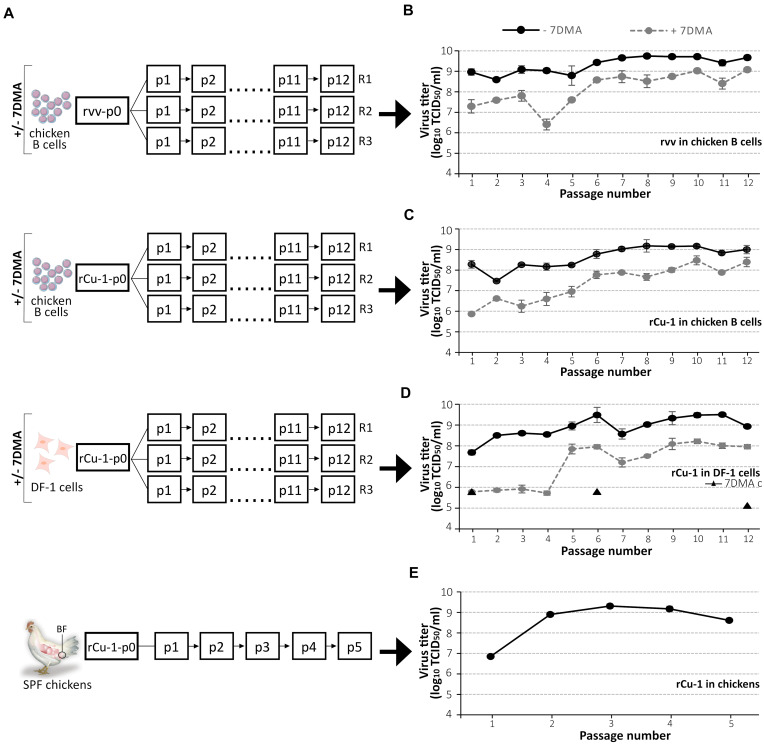
IBDV titer evolution during passages *in vitro*, *ex vivo*, and *in vivo*. **(A)** Layout of the serial passage strategy. For *in vitro* passages, a constant multiplicity of infection (MOI) of 0.01 and 0.1 was used in chicken B cells and DF-1 cells, respectively. For *ex vivo* and *in vitro* experiments, in chicken B cells and DF-1 cells, respectively, viral stocks from each passage were titrated in chicken B cells to adjust the infecting dose for the next passage. For *in vivo* experiments, five specific pathogen-free (SPF) chickens were inoculated with 10^5^ TCID_50_/animal in 0.1 mL PBS of rCu-1-p0 by the intranasal route. The five Bursae of Fabricius (BF) were collected 4 days post-inoculation, pooled and titrated in chicken B cells to adjust the infecting dose for the next passage. For each condition, each replicate of the final passage was sequenced by NGS (RNA deep-sequencing). Virus progeny titers after each of the 12 passages in cells in the presence or the absence of 7DMA treatment **(B–D)** or rCu-1 progeny titers after each of the 5 passages in chickens **(E)**. 7DMA c (triangle): control for constant activity of the drug 7DMA; DF-1 were infected with rCu-1-p0 in the presence of 7DMA in parallel of p1, p6, and p12. The anatomical position of the BF is indicated by a circle. p: number of passage; p0: inoculum; R: replicate. Bars indicate means ± SD based on three replicates for each passage and condition.

Serial passages of rCu-1-p0 in chickens were approved by ANSES ethical committee, registered at the national level under number C2EA-016/ComEth ANSES/ENVA/UPEC and authorized by French Ministry for higher education and research under permit number APAFiS#2494-201512021054584v1. For each passage, 5 five-to-eight-week-old SPF White Leghorns chickens (ANSES, Ploufragan, France) were housed in a single negative-pressure filtered-air isolators and infected by the intranasal route with rCu-1 at 10^5^ TCID_50_/animal in 0.1 mL PBS containing penicillin (200 IU/mL), streptomycin (0.2 mg/mL) and fungizone (2 μg/mL). At 4 days post-inoculation (d.p.i.), the five chickens were humanely euthanatized and BFs were collected aseptically, pooled and processed to prepare the viral stock for the next passage (see below). BFs were pooled in order to avoid creating a bias by selecting determined viral populations. After each passage, viral stock was titrated by ICC to adjust the dilution of the inoculum for the next passage and to infect at 10^5^ TCID_50_ per animal. A total of five passages in chickens were performed, similarly, to what is required by the European Pharmacopoeia to assess virus stability in the licensing process of IBDV live-attenuated vaccine-candidates. The codification for the different viral stocks is shown in Supplementary Table M1. A graphical overview of the different passaging procedures is presented in Figure 1A.

### Preparation of Viral Stocks From Bursae of Fabricius

Tissue homogenization was performed using either Ultra-Turrax T25 basic IKA or TissueLyser (Qiagen) homogenizers to process pool or individual BFs, respectively. All steps were carried out on ice. Briefly, bursae were weighed and cut into small segments. One milliliter of PBS was then added per gram of bursa before homogenizing. 1,1,1,2,3,4,4,5,5,5-Decafluoropentane (reference 94884, Sigma) was added to the tissue suspension (1:1 ratio) and an additional homogenization step was carried out. For Ultra-Turrax homogenizer procedure, the tissue suspension was spun at 1260 g for 30 min and supernatants were recovered. For TissueLyser homogenizer, tissue suspensions were shook at high-speed with a stainless steel bead for 3 min at 30 Hz, followed by centrifugation at 4000 g for 30 min. Supernatants were collected and stored at −70°C.

### Purification and Concentration of Viral Stocks

To purify rCu-1-p5/ch viral stock, the resin Capto^TM^Core 700 (reference 17548101, GE Healthcare), which retains molecules below 700 kDa and thus not viral capsids, was used as previously described (James et al., 2016). Three milliliters of viral stock were centrifuged for 10 min at 12000 g and supernatant was recovered and filtered through a 0.22 μm filter (Hall et al., 2014). The resin was washed 3 times with PBS, with shaking (5 min at low speed) followed by centrifugation (3 min at 800 g) in each washing step. Then, three sequential in-slurry purifications as previously described (James et al., 2016) were carried out in 15 mL tubes, using a ratio of five volumes of viral stock for each volume of resin. In each purification step, the mix was homogenized at low speed for 45 min on a rotator holder then centrifuged for 10 min at 800 g; supernatant was recovered and transferred into a new 15 mL tube containing resin. After the third purification, supernatants were subjected to concentration (see below). Viral stocks prepared from chicken B cells and DF-1 cells used for passage 12 of rCu-1 and from chicken B cells used for passage 12 of rvv (see above) were directly concentrated.

Supernatants were concentrated up to around 200 μL in Pierce^TM^ Protein Concentrator PES, 100K MWCO (reference 88524, Thermo Scientific) for 30 min at 3600 g and then stored at −70°C. All samples were then subjected to RNA extraction.

### RNA Extraction, Viral Genome RT-PCR, Gel Electrophoresis and Purification

One hundred and forty microliters from different viral stocks were RNA-extracted using the QIAamp viral RNA mini kit (reference 52904, Qiagen) following the manufacturer’s instructions. However, linear acrylamide (reference AM9520, Thermo Fisher) at 0.025 mg/mL was used instead of carrier RNA. RNA concentration was determined by using Qubit RNA HS assay kit (Invitrogen, Q32852) in the Qubit^®^2.0 Fluorometer.

The isolation of viral RNA from cloacal swabs was carried out using the NucleoMag^®^VET kit (reference 744200, Macherey Nagel) in the KingFisher^®^Flex system (reference 744950, Macherey Nagel), according to the manufacturer’s instructions. The obtained viral dsRNA was denatured in presence of DMSO at 94 °C for 4 min, then chilled on ice for 2 min. qRT-PCR was subsequently performed using QuantiTect Probe RT-PCR kit (reference 204443, Qiagen) according to the manufacturer’s protocol. Reactions were performed as follow: 30 min at 50 °C, 15 min at 95 °C, 40 cycles each comprising 15 s at 94 °C and 1 min 60 °C. The primers and probe used, which target a portion of VP3 coding region (CDR), were previously described (Escaffre et al., 2010).

Infectious bursal disease virus genome from individual BF or viral inocula used to infect chickens were reverse-transcribed into cDNA by Maxima H minus Reverse Transcriptase (reference EP0752, Thermo Fisher) according to the manufacturer’s protocol. The reaction was incubated at 50 °C for 30 min and heated at 85 °C for 5 min to inactivate the enzyme. cDNA was subjected for partial or full-segment PCR amplification with Phusion Hot Start II DNA polymerase (reference F549S, Thermo Fisher) following the manufacturer’s instructions. Reactions were performed as follow: 30 s at 98 °C, 35 cycles each comprising 10 s at 98 °C, 15 s at Tm, t at 72 °C and a final step at 72 °C for 5 min. Primer sets used during reverse-transcription and PCR, together with Tm and t for each PCR are presented in Supplementary Table 2. The segments A and B from each sample were separated by 1% agarose-gel electrophoresis, eluted in ultra-pure water by Gel and PCR clean up kit (reference 740609, Macherey Nagel). DNA concentrations were measured by using Qubit dsDNA HS assay kit (Invitrogen, Q32854) in the Qubit^®^2.0 Fluorometer.

### Study of Pathogenicity in Chickens

Pathogenicity assessment in SPF chickens was approved by ANSES ethical committee, registered at the national level under number C2EA-016/ComEth ANSES/ENVA/UPEC and authorized by French Ministry for higher education and research under permit number APAFiS#4945-20 16041316546318 v6. Eighty, three-week-old SPF White Leghorns chickens (ANSES, Ploufragan, France) were distributed into 4 groups of 20 birds, named P1, P2, P3, and P4, of similar weight and sex, housed in separate negative-pressure filtered-air isolators. Three days before inoculation, blood samples were collected from 27 chickens in order to confirm their seronegativity for IBDV using a viral neutralization assay as previously described (Eterradossi et al., 1992). Viral inocula were prepared by diluting viral stocks in a diluent made of PBS with penicillin (200 IU/mL), streptomycin (0.2 mg/mL) and fungizone (2 μg/mL). Chickens from groups P1, P2 and P3 were inoculated by the intranasal route with rvv-p0, rvv-p12 or rvv-p12/7DMA, respectively, at 10^6^ TCID_50_ in 0.1 mL per animal. Chickens from group P4 were mock-inoculated with diluent.

Mortality rates were followed throughout the animal experiment, which lasted for 21 days. Clinical signs were measured daily based on the symptomatic index previously developed which ranges from 0, “lack of signs” to 3, “typical severe IBD signs with prostration or death,” which is the ethical endpoint (Le Nouen et al., 2012). Cloacal swabs were collected in 1 mL PBS at 0–4, 7, 14, and 21 d.p.i. to measure viral shedding by qRT-PCR (see section 2.7).

Ten randomly chosen chickens were blood sampled at the venous occipital sinus using commercial EDTA coated blood collection devices (Monovette^®^, Sarstedt) at 2 and 21 d.p.i to perform white blood cell counting as described in 2.9.

At 4 d.p.i. and 21 d.p.i., five chickens per group and all remaining chickens, respectively, were weighed, humanely euthanatized, necropsied and their spleens and BFs were collected and weighed for calculating the spleen-to-body-weight ratio [s/B] and the bursa-to-body-weight ratio [b/B], respectively. Then, the bursae harvested at 4 d.p.i. were processed for virological analyses as indicated above (see section 2.5) for virus titration (see section 2.3).

### Flow Cytometry

The protocol for chicken white blood cells counting was performed according to Seliger et al. (2012). All blood samples were processed within 4 h after blood collection. Briefly, EDTA-blood samples were diluted in PBS with 1% FBS, mixed with the labeled antibody mixture and incubated for 30 min with agitation, at room temperature and in the dark. The antibodies and fluorochrome conjugates used in this study were similar to those used by Seliger et al. (2012) and they are listed in Supplementary Table 3. The gating strategy is showed in Supplementary Figure 1. After incubation, approximately 5 × 10^4^ Precision Count Beads (reference BLE424902, BioLegend) prepared in PBS-FBS were added to each sample to determine the absolute counts of cells. In the last step, formaldehyde (1% final concentration per sample) was added and the samples were incubated for 15 min with frequent agitation at room temperature and in the dark to inactivate the virus. Samples were analyzed on a FC500 MPL flow cytometer (Beckman Coulter).

### Sequencing of Genomic Segments by Next Generation Sequencing (NGS)

To quantify IBDV evolution, RNA molecules were extracted from rvv-p0 and rCu-1-p0, all stocks obtained after passage 12 in chicken B cells and DF-1 cells and after passage 5 in chickens. These RNAs were then analyzed by a next generation sequencing (NGS) approach.

For RNA sequencing, NGS was performed on the RNA extract after rRNA depletion with the Low Input Ribominus Kit (Ambion), as described by the manufacturer. A RNA library was obtained using Ion total-Seq Kit v2 (Life Technologies) according to the manufacturer’s recommendations and was then sequenced with using Ion Torrent Proton technology.

For DNA sequencing, PCR fragments were purified and quantified as described above. Illumina libraries were prepared with the Nextera XT kit (Illumina) according to the supplier’s recommendation. Paired-end sequencing (150 nt long) was performed on an Illumina NovaSeq 6000.

All sequencing reads were processed to remove low quality or artefactual reads followed by bioinformatics analysis as previously described (Abed et al., 2018). The data were deposited in the BioSample database at NCBI under accession numbers from SAMN14642839 to SAMN14642909.

An in-house bioinformatics pipeline, VVV (Flageul et al., 2021) was used to identify single nucleotide variation (SNV) frequencies above an empirically determined cut-off frequency of 7% to exclude the errors introduced by reverse-transcription, PCR and sequencing. Sequences of the PCR primers (Supplementary Table 2) as well as nucleotide substitutions in homopolymer regions and insertions and deletions were removed from the analysis (Song et al., 2017). The total variants which passed these criteria were used to calculate the nucleotide diversity (π), which allows to measure viral sequence diversity without bias due to the depth of sequencing. At the locus l, n_*i*_ (copies of the allele i in the A, T, C, G set) was calculated as n_*i*_ = p_*i*_/N and n_*j*_ = 1-n_*i*_ where N is the read depth at the locus l and the frequency of each nucleotide i was given by p_*i*_. Then, the proportion of pairwise differences between alleles can be denoted Dl (Zhao and Illingworth, 2019):

$$Dl=\frac{\sum_{i\neq j}n_{i}n_{j}}{\frac{1}{2}N\left(N-1\right)}=\frac{N\left(N-1\right)-\sum_{i}n_{i}\left(n_{i}-1\right)}{N\left(N-1\right)}$$

Therefore, the statistic π was calculated for all covered positions in each genomic segment (L) as,

$$\pi =\sum_{l=1}^{L}Dl/L$$

To render a more user-friendly number, π values were multiplied by 10 000.

### Prediction of RNA Secondary Structures of rvv-p0, rvv-p12, rCu-1-p0, and rCu-1-p12/B and Detection of Mutations Present in Other Naturally Occurring IBDV Strains

RNA secondary structures were predicted using mFold_util version 4.7 online tool (Zuker, 2003). Complete or nearly complete sequences of segments A and B of IBDV were downloaded from Genbank database^1^. Sequences with too many ambiguous nucleotide positions were removed. Amino acid sequences for each viral protein were aligned using MAFFT program. After alignment, sequences of vaccine or laboratory-derived strains were excluded from the analysis.

### Statistical Analysis

Statistical analyses were performed using R version 3.6.1 and RStudio Version 1.2.5019. Differences in mortality were analyzed by logistic regression using the “glm” function from the package “stats” version 3.6.1., associated with a binomial link. All other quantitative parameters were analyzed using Kruskal-Wallis test followed by Fisher’s least significant difference test with Holm adjustment method for multiple comparisons using the “kruskal” function from the package “Agricolae” version 1.3–2.

## Results

### Viral Titer Evolution During Passages in Cells and Chickens

To help to understand the different molecular mechanisms that drive viral adaptation and evolution in different environments, rCu-1-p0 and rvv-p0 were passaged 12 times in the absence and presence of 7DMA with a defined MOI in chicken B cells. Additionally, the genetic stability of rCu-1-p0 genome upon passages *ex vivo* in chicken B cells was compared to its passages *in vitro* in DF-1 cells and *in vivo* in chickens. As rvv is not adapted to cell culture in DF1, this virus was not passaged on this cell line; this virus was also not passaged in chickens.

Figure 1A shows the layout followed during the different passages. In the absence of 7DMA and in DF-1 and chicken B cells, rvv and rCu-1 titers showed little variation, about 0.2–0.7 log_10_ (TCID_50_/mL), between one passage and the next (Figures 1B–D). Passaging of rCu-1 in chickens resulted in an increase of around 2 log_10_ (TCID_50_/mL) between p1 and p2; virus yield remained similar after p2 (Figure 1E). No clinical sign was observed during the *in vivo* passages of rCu-1 although bursae were collected 4 days post-infection, a moment when clinical signs usually reach their maximum for pathogenic IBDV strains.

During the first passage, 7DMA treatment provoked, in agreement with preliminary tests, a mean reduction of rvv titer of 1.7 log_10_ (TCID_50_/mL) in chicken B cells and, in rCu-1, a mean reduction of 2.4 and 1.9 log_10_ (TCID_50_/mL) in chicken B cells and DF-1 cells, respectively. In presence of 7DMA, titers were notably lower in DF1 cells in early passages (1–4) (around 6 log_10_ (TCID_50_/ml)) compared to those of the same viruses passaged in the absence of 7DMA (around 8.5 log_10_ (TCID_50_/ml)). However, after passage 4 and in presence of 7DMA, these increased to around 7.8 log_10_ (TCID_50_/ml), which was within 1 to 1.5 log_10_ (TCID_50_/ml) of titers obtained for the same viruses passaged in the absence of 7DMA (Figure 1D). A similar effect in passages 1–5 was observed in chicken B cells for both rCu-1 and rvv; however, this was less marked (Figures 1B,C).

As a control for 7DMA activity, DF-1 were infected with rCu-1-p0 in the presence of 7DMA in parallel of passages 1, 6 and 12. The resulting viral titers, of 5.8 log_10_ (TCID_50_/mL) in p1, 5.8 log_10_ (TCID_50_/mL) in p6 and 5.1 log_10_ (TCID_50_/mL) in p12, similar to those observed in initial passages upon 7DMA treatment, showed constant activity of the drug 7DMA (Figure 1D, triangle).

Collectively, data indicated an increased resistance of the IBDV strains passaged in the presence of 7DMA, possibly associated to some event of mutation after passage 4 or 5.

### Variant Frequency, Mutation Frequency and Nucleotide Diversity (π) of rvv and rCu-1 After Passages

To quantify IBDV evolution, RNA molecules were extracted from rvv-p0 and rCu-1-p0, all stocks obtained after passage 12 in chicken B cells and DF-1 cells and after passage 5 in chickens. These RNAs were then analyzed by NGS. Assembled sequences were further analyzed to calculate the variant frequency, mutation frequency and nucleotide diversity (π). The frequency and distribution of SNVs are presented in Tables 1–3. For clarity sake, only mutation frequency and nucleotide diversity values are in detail below.

**TABLE 1:**
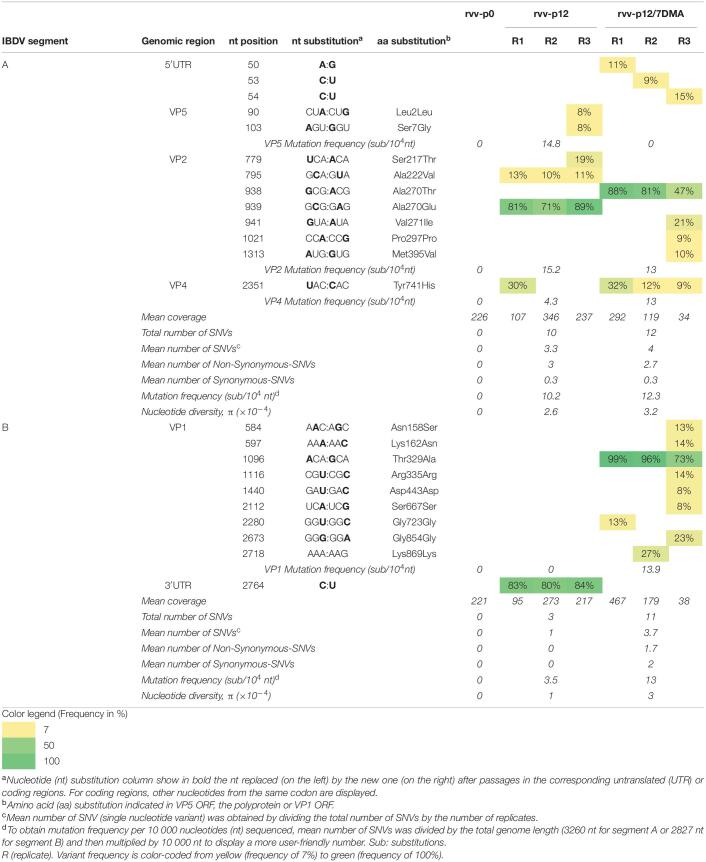
Analysis of variant frequencies and genetic variation in IBDV genome of rvv-p0, rvv-p12, and rvv-p12/7DMA characterized by RNA deep-sequencing.

**TABLE 2:**
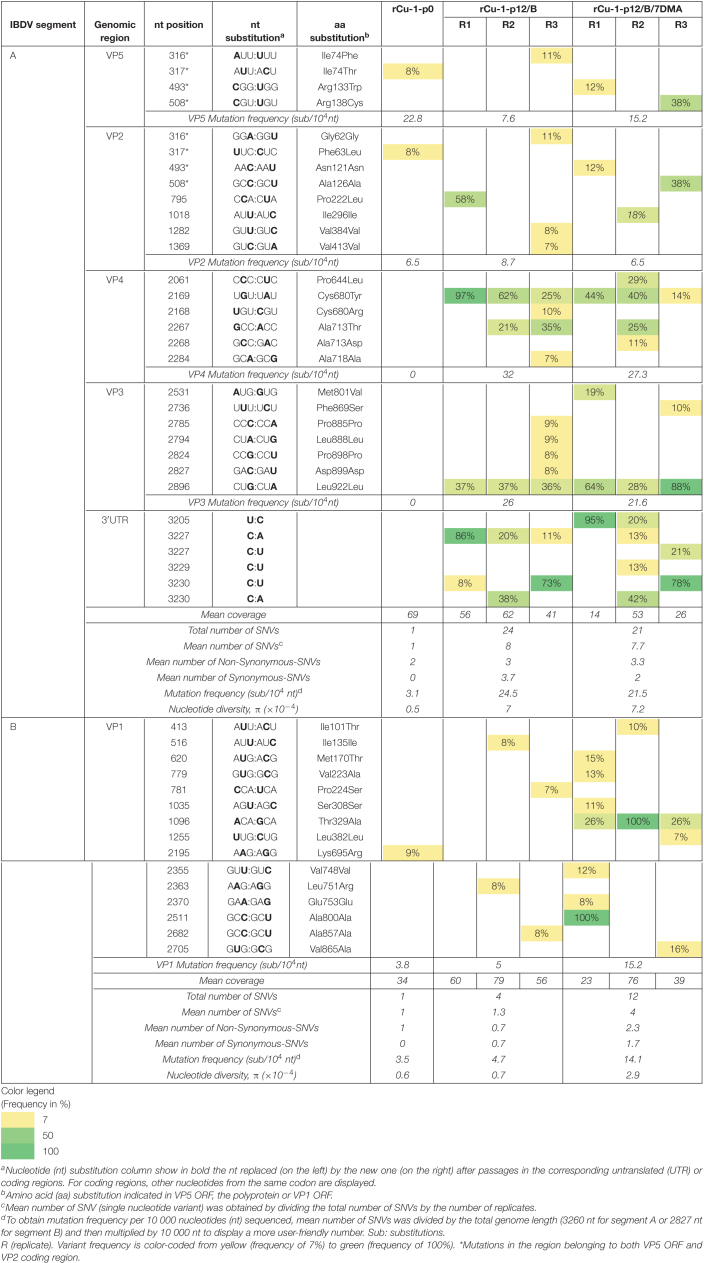
Analysis of variant frequencies and genetic variation in IBDV genome of rCu-1-p0, rCu-1-p12/B, and rCu-1-p12/B/7DMA characterized by RNA deep-sequencing.

**TABLE 3:**
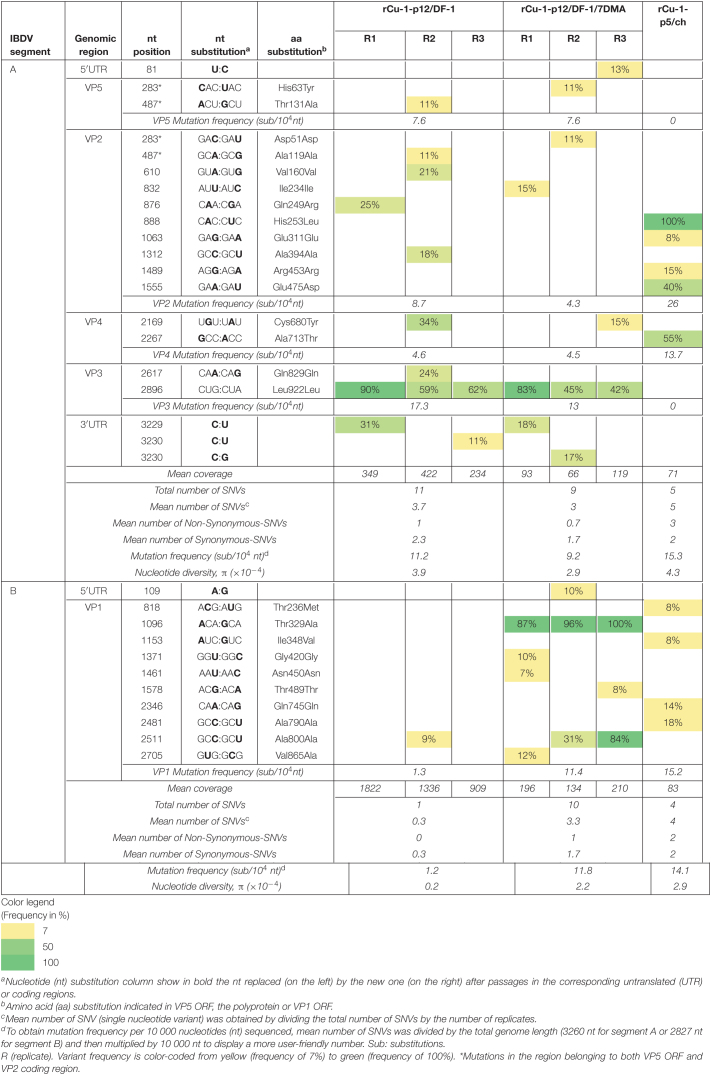
Analysis of variant frequencies and genetic variation in IBDV genome of rCu-1-p12/DF-1, rCu-1-p12/DF-1/7DMA, and rCu-1-p5/ch characterized by RNA deep-sequencing.

#### rvv in Chicken B Cells

No SNV was observed in rvv-p0 segment A nor B viral RNA population (Table 1) compared to the consensus sequence of the plasmid inserts sequences of rvv.

##### Analysis of segment A of rvv-p12 and rvv-p12/7DMA

For rvv-p12, mutation frequencies of 14.8, 15.2, and 4.3 substitutions per 10 000 nucleotides (sub/10^4^ nt) were observed for VP5, VP2 and VP4, respectively.

For rvv-p12/7DMA, mutation frequency in VP2 (13 sub/10^4^ nt) was similar to that in rvv-p12, while this frequency was 3 times higher in VP4 CDR in rvv-p12/7DMA (13 sub/10^4^ nt) compared to that in rvv-p12.

In segment A, the mutation frequency as well as the nucleotide diversity, determined through the statistic π were comparable for both viral stocks (Table 1).

##### Analysis of segment B of rvv-p12 and rvv-p12/7DMA

For rvv-p12, 3 SNVs were found (Table 1), located in the 3′UTR in one single nucleotide position.

For rvv-p12/7DMA, 11 SNVs were found (Table 1), all of them located in VP1 ORF in 9 nucleotide positions, with a mutation frequency of 13.9 sub/10^4^ nt.

The mutation frequency increased from 3.5 sub/10^4^ nt in rvv-p12 to 13 sub/10^4^ nt in rvv-p12/7DMA. Unlike segment A, in segment B, the nucleotide diversity increased 3 times in rvv-p12/7DMA (3 × 10^–4^) compared with rvv-p12 (1 × 10^–4^, Table 1).

#### rCu-1 in Chicken B Cells, DF-1 and Chickens

rCu-1-p0 harbored a single SNV in segment A (in the region belonging to both VP5 ORF and VP2 CDR) and another one in segment B, with frequencies below 10% (Table 2).

After 12 passages in chicken B cells and DF-1 as well as after 5 passages in chickens of rCu-1-p0, several SNVs were distributed throughout the genome in coding as well as non-coding regions in both segments regardless of the condition used.

##### Analysis of segment A from rCu-1-p12/B and rCu-1-p12/B/7DMA

In rCu-1-p12/B, mutations frequencies of 7.6, 8.7, 32, and 26 sub/10^4^ nt were observed for VP5, VP2, VP4 and VP3, respectively. In rCu-1-p12/B/7DMA, mutation frequencies of 15.2, 6.5, 27.3, and 21.6 sub/10^4^ nt were observed for VP5, VP2, VP4 and VP3, respectively. In segment A, the mutation frequency as well as the nucleotide diversity were comparable in both viral stocks (Table 2).

##### Analysis of segment B from rCu-1-p12/B and rCu-1-p12/B/7DMA

In rCu-1-p12/B, a mutation frequency of 5 sub/10^4^ nt was observed in VP1 ORF. This frequency increased 3 times rCu-1-p12/B/7DMA.

For segment B, in rCu-1-p12/B, a mutation frequency of 4.7 sub/10^4^ nt and a nucleotide diversity of 0.7 × 10^–4^ were observed. In rCu-1-p12/B/7DMA, the mutation frequency and the nucleotide diversity increased 3 and 4.1 times, respectively, with respect to rCu-1-p12/B.

##### Analysis of segment A from rCu-1-p12/DF-1 and rCu-1-p12/DF-1/7DMA

In rCu-1-p12/DF-1, mutation frequencies of 7.6, 8.7, 4.6, and 17.3 sub/10^4^ nt were observed for VP5, VP2, VP4 and VP3, respectively (Table 3).

In rCu-1-p12/DF-1/7DMA, comparable mutation frequencies with respect to rCu-1-p12/DF-1 were observed in VP5, VP3 and VP4. In contrast, a two-fold increase was apparent in rCu-1-p12/DF-1 VP2 CDR compared with to rCu-1-p12/DF-1/7DMA (Table 3).

In this segment, the mutation frequency and the nucleotide diversity (Table 3) were comparable for both viral stocks.

##### Analysis of segment B from rCu-1-p12/DF-1 and rCu-1-p12/DF-1/7DMA

In rCu-1-p12/DF-1, the observed mutation frequency for VP1 ORF was 1.3 sub/10^4^ nt (Table 3), while in rCu-1-p12/DF-1/7DMA, this frequency increased 9 times (11.4 sub/10^4^ nt). For segment B, in rCu-1-p12/DF-1/7DMA, the mutation frequency and the nucleotide diversity (Table 3) increased 9.8 and 11 times, respectively, in comparison with rCu-1-p12/DF-1.

##### Analysis of segments A and B from rCu-1-p5/ch

In segment A, mutation frequencies of 26 and 13.7 sub/10^4^ nt were observed for VP2 and VP4, respectively. In segment B, the mutation frequency reached a value of 15.2 sub/10^4^ nt. The mutation frequency as well as the nucleotide diversity (Table 3) were comparable between both segments.

The results in section 3.2. indicated that although serial passages were associated with the appearance of mutations in both segments, 7DMA seemed to specially target the viral polymerase VP1 as previously described in other viruses (Olsen et al., 2004; Zmurko et al., 2016), producing a higher increase in the number of mutations and nucleotide diversity in segment B than in segment A.

### Dominant Mutations After Serial Passaging

In order to evaluate the relevance of chicken B cells as a cell system for IBDV replication and study the adaptability of virus to new conditions such as the presence of 7DMA, dominant mutations (that appeared at consensus levels, i.e., with frequencies higher than 50%) detected after passages in chickens and cells were further analyzed. To guide the reader, mutations in VP2, VP4 and VP3 CDRs are indicated as substitutions along the amino acid numbering of the polyprotein.

#### rvv in Chicken B Cells

##### Dominant mutations in segment A from rvv-p12 and rvv-p12/7DMA

In rvv-p12, only one dominant mutation, observed in all replicates, was identified in VP2 CDR, inducing the Ala270Glu change (Table 1). In rvv-p12/7DMA, one dominant mutation was identified in all replicates in the same position 270, although that change encoded a threonine instead of a glutamic acid (Ala270Thr, Table 1). No mutation previously associated to cell culture adaptation, such as changes in VP2 positions 253, 279, 284 or 330, was detected in neither rvv-p12 nor rvv-p12/7DMA.

##### Dominant mutations in segment B from rvv-p12 and rvv-p12/7DMA

**FIGURE 2:**
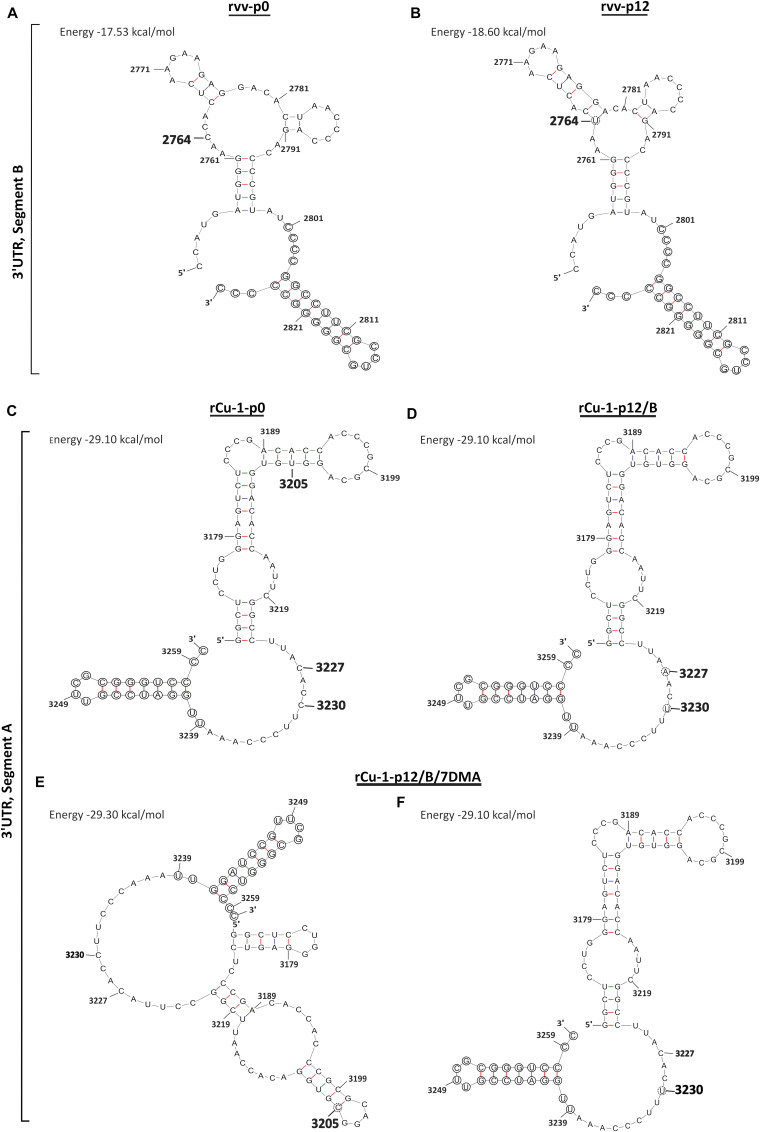
Predictions of RNA secondary structures in the 3′UTR in IBDV genome. **(A)** The original 3′UTR sequence of segment B in rvv-p0 featuring in bigger bold number the mutated nucleotide position after passaging. **(B)** The 3′UTR sequence of segment B in rvv-p12, featuring in a dotted circle the nucleotide substitution. **(C)** The original 3′UTR sequence of segment A in rCu-1-p0 featuring in bigger bold numbers the mutated nucleotide positions after passaging. **(D)** The 3′UTR sequence of segment A in rCu-1/p12/B, featuring in a dotted circle the nucleotide substitution. **(E,F)** The 3′UTR sequence of segment A in rCu-1-p12/B/7DMA, featuring in dotted circles the nucleotide substitutions. In circles, conserved regions in 3′UTR sequences. Energy levels for each structure are given in kcal/mol in brackets.

In rvv-p12, one dominant mutation located in the nucleotide position 2764 (Table 1) was detected in the 3′UTR. Since the UTRs play an important role during the replication and translation of IBDV (Boot and Pritz-Verschuren, 2004), their secondary structures were analyzed. The mutation in nucleotide position 2764 showed a branch stem-loop structure as well as in rvv-p0 (Figures 2A,B). Although RNA structure prediction revealed a conformational change, where the mutation C2764U provoked an extension in the stem located in the stem-loop into 2767–2777 region (Figure 2B), both structures conserved the stem-loop located into the 2801–2827 region previously described (Boot and Pritz-Verschuren, 2004).

In rvv-p12/7DMA, a single dominant mutation in VP1 ORF was observed in all replicates, inducing the Thr329Ala change (Table 1).

#### rCu-1 in Cells and Chickens

##### Dominant mutations in segment A from rCu-1-p12/B and rCu-1-p12/B/7DMA

In rCu-1-p12/B, no SNVs was dominant in all three replicates, however, dominant SNVs were observed in certain replicates (Table 2). A dominant mutation in VP2 CDR and another in VP4 CDR were observed, resulting in the Pro222Leu and Cys680Tyr mutations, respectively (Table 2). Two dominant SNVs were located in 3′UTR, resulting in the C3227A and C3230U mutations (Table 2), although they did not produce a conformational change in the 3′UTR structure, which showed a branch stem-loop structure as well as in rCu-1-p0 (Figures 2C,D). In this prediction, the two-nucleotide substitutions, C3227A and C3230U, present in rCu-1-p12/B genome, were supposed to coexist in the same RNA molecule due to their, similarly, high frequency in two given replicates (R1 and R3, Table 2).

In rCu-1-p12/B/7DMA, the dominant, synonymous mutation Leu922Leu was detected in VP3 CDR (Table 2). Two dominant SNVs were located in 3′UTR, resulting in the U3205C and C3230U mutations: since none of those mutations coexisted in any given replicate, each mutation was considered in a distinct RNA molecule for RNA secondary structure determination. The mutation U3205C provoked the apparition of an extra stem-loop into the 3170–3183 region (Figure 2E), while C3230U did not modify the RNA structure with respect to the structure shown by rCu-1-p0 (Figure 2C). All these structures (Figures 2C–F) have in common that they conserve the stem-loop located into the 3239–3260 region, as previously described (Boot and Pritz-Verschuren, 2004). For each prediction, two additional structural variations were computed as possible: all the predicted structures displayed the same branch stem-loop structure, therefore only the most thermodynamically stable structures are presented.

##### Dominant mutations in segment B from rCu-1-p12/B and rCu-1-p12/B/7DMA

No dominant mutation was detected in rCu-1-p12/B. However, the 7DMA treatment induced the Thr329Ala and Ala800Ala mutations in VP1 ORF. Although Thr329Ala emerged as a dominant change, with a frequency of 100%, it was only observed in one replicate (Table 2).

##### Dominant mutations in segment A from rCu-1-p12/DF-1 and rCu-1-p12/DF-1/7DMA

Only the Leu922Leu synonymous mutation in VP3 CDR reached consensus levels in rCu-1-p12/DF-1 and rCu-1-p12/DF-1/7DMA (Table 3).

##### Dominant mutations in segment B from rCu-1-p12/DF-1 and rCu-1-p12/DF-1/7DMA

No dominant mutation was detected in rCu-1-p12/DF-1. In rCu-1/DF-1/7DMA, the Thr329Ala change was observed in all replicates, while Ala800Ala change was present in one replicate (Table 3).

##### Dominant mutations in segment A and B from rCu-1-p5/ch

In segment A, two dominant mutations were detected, one in hVP2 (His253Leu), with 100% of variant frequency and the second one in VP4 CDR (Ala713Thr), with 55% variant frequency (Table 3). The latter was also detected in two replicates in rCu-1-p12/B but at frequencies lower than 50% (21 and 35%, respectively, Table 3). No dominant mutation was detected in segment B.

The results in section 3.3 showed that few dominant mutations were detected after passages in non-coding regions together with non-synonymous mutations in VP2 (positions 222, 253 and 270) and in VP4 (positions 680 and 713) CDRs. In addition, the mutation Thr329Ala in VP1 ORF specifically appeared after 7DMA treatment in both cell types and IBDV strains.

### Pathogenicity of rvv-p0, rvv-p12, and rvv-p12/7DMA in SPF Chickens

**FIGURE 3:**
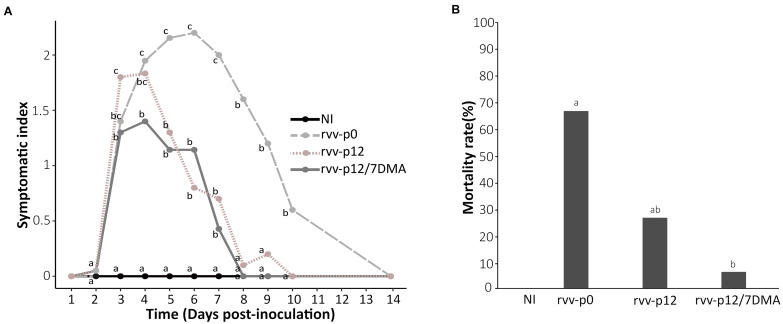
Analysis of pathogenicity in SPF chickens infected with rvv-p0, rvv-p12, and rvv-p12/7DMA. **(A)** Mean symptomatic index between 1 and 14 day post-inoculation (d.p.i.) in chickens infected with rvv-p0, rvv-p12, or rvv-p12/7DMA. This index ranges from 0, “lack of signs”; 1, “typical IBD signs with ruffled feathers; motility is not reduced”; 2, “typical IBD signs with dehydration; motility slightly reduced”; to 3, “typical severe IBD signs (ethical endpoint) with prostration or death.” **(B)** Cumulated mortality rate at 21 d.p.i. in chickens infected with rvv, rvv-p12 or rvv-p12/7DMA. Different letters indicate a *p*-value < 0.05 between the different groups using logistic regression. NI, non-infected group.

In order to get further insight into the impact of the mutant spectra obtained after passaging on virulence, the pathogenicity of the stocks of rvv-p12 or rvv-p12/7DMA was assessed in SPF chickens and compared with rvv-p0, which is a genetically homogenous inoculum. For this purpose, among three replicates for each condition, the replicate with a representative number of variants and dominant mutations was selected (Table 1). Accordingly, replicates R1 for both rvv-p12 (with median percentages for the dominant mutations Ala270Glu in VP2 and C2764U in 3′UTR in segment B) and rvv-p12/7DMA (with the highest variant frequency for the mutation Thr329Ala in VP1, 99%) were chosen to carry out the animal trial in SPF chickens using the standard protocol previously described (Le Nouen et al., 2012). Neither clinical signs nor mortality were observed in non-infected control chickens (NI, Figure 3A). In contrast, inoculation with rvv-p0 and rvv-p12 resulted in mild to severe clinical signs, characterized by diarrhea, prostration and ruffled feathers, with a mean symptomatic index of 1.95 and 1.83 at 4 d.p.i., inducing 67 and 27% of mortality, respectively (Figure 3B). In contrast, rvv-p12/7DMA resulted in 7% of mortality associated with mild clinical signs and a mean symptomatic index of 1.4 at 4 d.p.i. The statistical analysis showed that although the mortality induced by rvv-p0 was higher than that induced by rvv-p12, it did not differ significantly. In contrast, the mortality rate produced by rvv-p0 differed significantly from the mortality induced by rvv-p12/7DMA, indicative of attenuation of the later *in vivo*.

**FIGURE 4:**
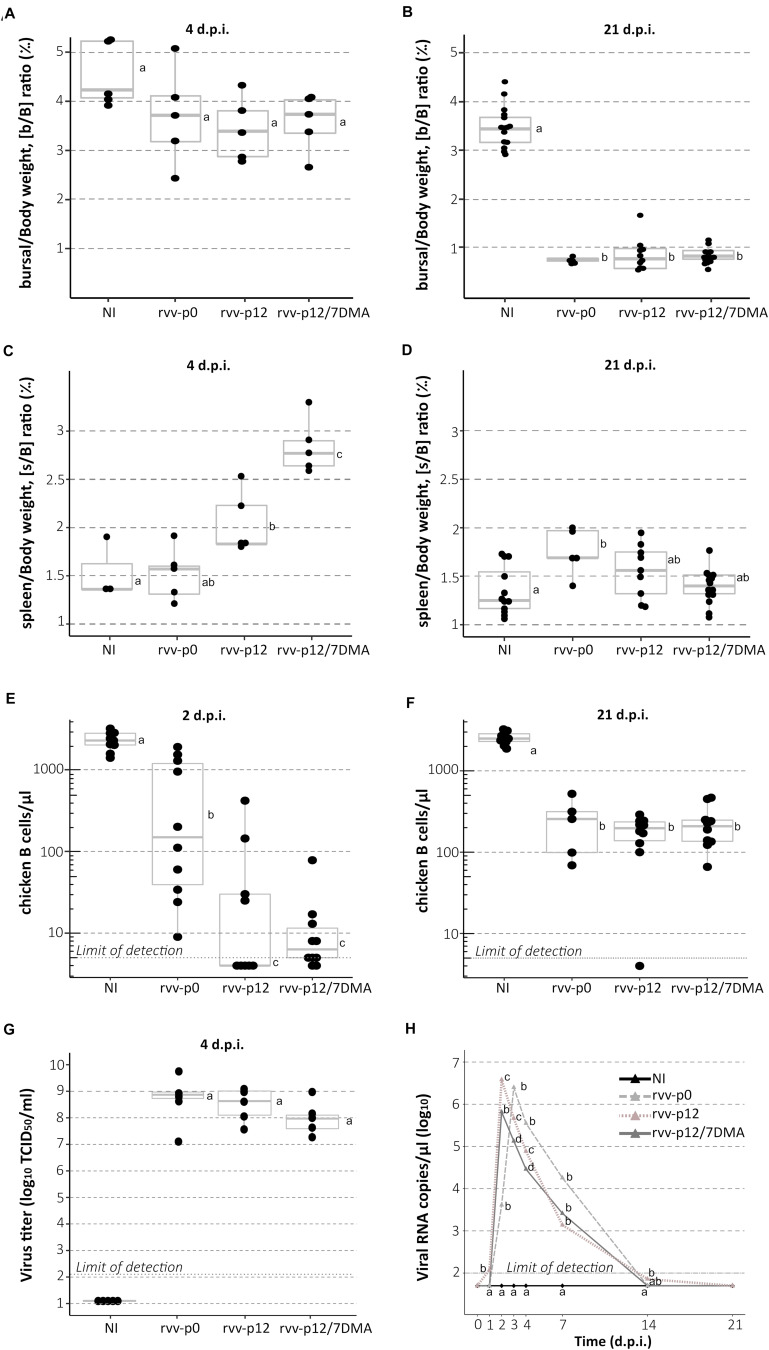
Bursal-to-body weight ratio, spleen-to-body-weight ratio, chicken B cells counting, viral load in Bursa of Fabricius and viral shedding in chickens infected with rvv-p0, rvv-p12, or rvv-p12/7DMA. **(A,B)** Bursal-to-body weight [b/B] ratio (‰) at 4 (*n* = 5 chickens per group) and 21 days (at least 5 chickens per group) post-inoculation (d.p.i.). **(C,D)** Spleen-to-body-weight [s/B] ratio (‰) at 4 (*n* = 5 chickens per group) and 21 d.p.i. (at least 5 chickens per group). **(E,F)** Chicken B cell concentration in blood at 2 and 21 d.p.i. (*n* = 10 chickens per group except for the rvv-p0 group). **(G)** Virus titer at day 4 in Bursa of Fabricius [in log_10_ (TCID_50_/mL), *n* = 5 chickens per group]. For **A–G**, points represent individual values; boxplots indicate median and interquartile range. **(H)** Median viral shedding per group (in viral RNA copies/μL) at day 0–4, 7, 14, and 21 from cloacal swabs. For all graphs, different letters indicate a *p*-value < 0.05 between the different groups using Kruskal-Wallis test followed by Fisher’s least significant difference test with Holm adjustment method for multiple comparisons. NI, non-infected group.

To study bursal atrophy, gross changes in spleen and chicken B cell depletion, analysis of bursal-to-body weight [b/B] ratio, spleen-to-body-weight ratio [s/B] and counting of blood cells were carried out, respectively. b/B ratio did not reveal any significant differences at 4 d.p.i (Figure 4A) between the four different groups. However, b/B ratio showed marked bursal atrophy at 21 d.p.i in all three challenged groups, with an average 4.4 fold reduction in comparison with mock-infected birds (Figure 4B). In contrast, an increase was globally observed for s/B in infected groups (Figures 4C,D): this increase was not statistically significant for the rvv-p0 group (median value: 1.57 ‰ versus 1.36 ‰ for NI), unlike the rvv-p12 and rvv-p12/7DMA groups; for the latter, marked splenomegaly was observed with a median value of 2.77‰. Splenomegaly was no longer visible at 21 d.p.i. except for the rvv-p0 group (Figure 4D).

To count the number of circulating chicken B cells, blood samples were taken from chickens in each group. In general, chicken B cells depletion was observed in all the infected groups (Figures 4E,F). At 2 d.p.i., a reduced chicken B cell concentration was found in birds from the rvv-p0 group (median cell count of 155.5/μL) in comparison with birds from NI (median cell count of 2279/μL). This concentration appeared significantly higher than that of birds from rvv-p12 (median cell count below the detection limit of 5/μL) and rvv-p12/7DMA (median cell count of 6.5/μL) groups (Figure 4E). At 21 d.p.i, a comparable recovery in the number of chicken B cells was observed in the three infected groups, although all of them appeared 10-fold below the concentration of B cells detected in the NI group (Figure 4F). A similar pattern of early reduction compared with NI birds was observed for other blood cell types, with mild cell-type specific variations (Supplementary Figure 2). Namely, for T cells and thrombocytes, a significant reduction was apparent in all groups at 2 d.p.i. At the same time, a reduction in the number of monocytes was only significant for rvv-p12 and rvvp12/7DMA groups. At 21 d.p.i., cells numbers from infected groups tended to go back to values closer to that of control birds.

To determine the viral load in BF and to monitor cloacal viral shedding, viruses from bursae were biologically titrated and viral RNA from cloacal swabs were quantified. High and similar bursal viral titers were detected among the three challenged groups at 4 d.p.i. (Figure 4G). In general, the cloacal viral excretion of rvv-p12/7DMA, measured by qRT-PCR, remained lower than those found in the other two rvv groups, reaching a reduction of 1 log_10_ (copies/μL RNA) at 3 and 4 d.p.i compared to the rvv-p0 group (Figure 4H). rvv-p12 viral shedding was similar to that detected in the rvv-p0 group. Although the three challenge viruses seem to display similar replication in the BF, these results collectively indicate that rvv-p12/7DMA, unlike rvv-p12, shows a reduced pathogenicity in comparison with the parental rvv strain.

### Variant Frequency, Dominant Mutations, Mutation Frequency and Nucleotide Diversity (π) in rvv-p0/BF, rvv-p12/BF, and rvv-p12/7DMA/BF

In order to characterize the viral population during infection after inoculation with rvv-p0, rvv-p12 and rvv-p12/7DMA, viral RNA were extracted from the BF sampled at 4 d.p.i. of chicken inoculated with these viruses. These viral RNA were reverse transcribed, both genomic segments A and B were PCR-amplified and subsequently subjected to DNAseq together with the inoculum of each group. Complete PCR amplification of IBDV genome did not work, neither with viral inocula nor with some samples from BF (Table 4 shows for which samples complete genome RT-PCR was successful and which ones were used to analysis). For those samples, partial genome RT-PCR was attempted instead, producing amplicons that spanned the regions where dominant mutations were detected by RNA deep-sequencing. With this second-choice strategy, amplification was successful for the three inocula and failed again for the bursa-derived samples.

**TABLE 4:**
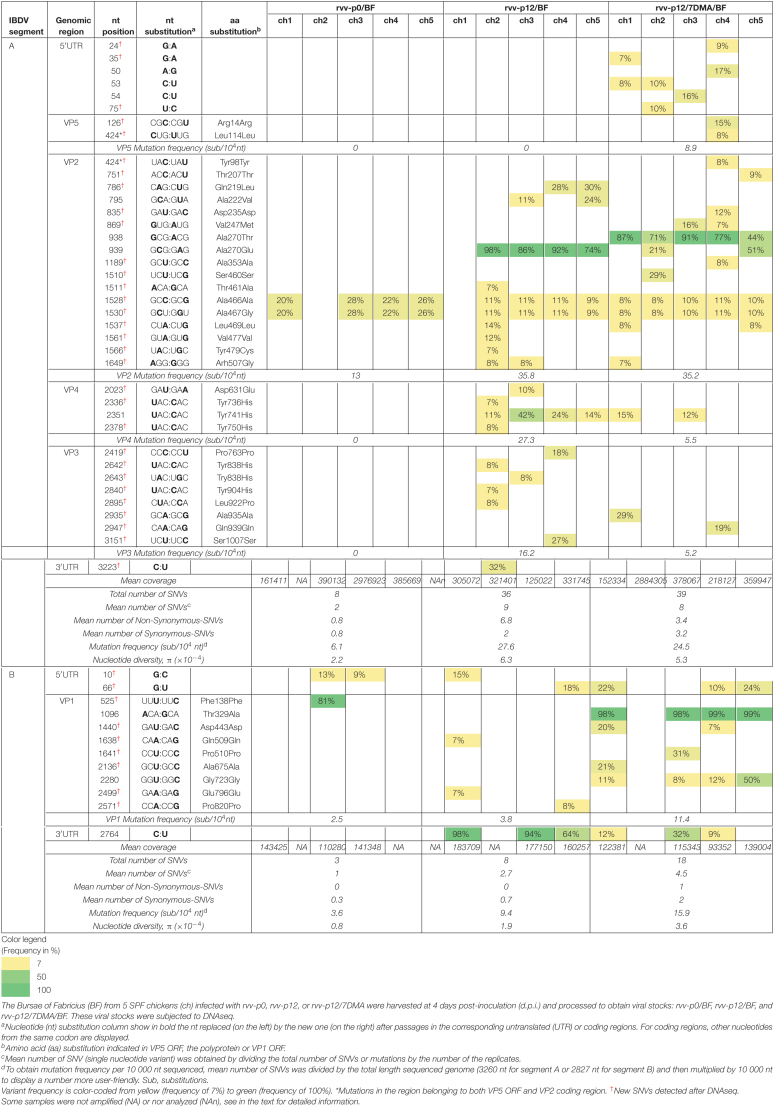
Analysis of variant frequencies and genetic variation in IBDV genome of rvv-p0, rvv-p12, and rvv-p12/7DMA recovered from Bursa of Fabricius.

Similar number of SNVs and variant frequencies were detected between rvv-p0, rvv-p12 and rvv-p12/7DMA inocula sequenced by RNA deep-sequencing and DNAseq (Table 1 and Supplementary Table 2, respectively). To avoid bias, mutation frequency and nucleotide diversity parameters presented in Table 4 were normalized to the number of samples and the length of region sequenced.

#### Analysis of Segment A From rvv-p0/BF, rvv-p12/BF, and rvv-p12/7DMA/BF

No mutation was found in rvv-p0 inoculum (Supplementary Table 4) while eight new SNVs were detected in VP2 CDR in rvv-p0/BF, distributing in two nucleotide positions (Table 4). VP2 CDR reached a mutation frequency of 13 sub/10^4^ nt.

New SNVs were detected in rvv-p12/BF with respect to rvv-p12 (Table 4), distributing in eight nucleotide positions in VP2 CDR, three in VP4 CDR, six in VP3 CDR and one in the 3′UTR. VP2, VP4 and VP3 reached mutation frequencies of 35.8, 27.3, and 16.2 sub/10^4^ nt, respectively.

New SNVs emerged in rvv-p12/7DMA/BF with respect to rvv-p12/7DMA (Table 4), distributing in three nucleotide positions in the 5′UTR, one in VP5 ORF, one in in the region belonging to both VP5 ORF and VP2 CDR, nine in VP2 CDR and two in VP3 CDR. VP5, VP2, VP4 and VP3 reached mutation frequencies of 8.9, 35.2, 5.5, and 5.2 sub/10^4^ nt, respectively.

Concerning dominant mutations, only two, Ala270Glu or Ala270Thr, were detected in VP2 CDR in rvv-p12/BF or rvv-p12/7DMA/BF, respectively, in most of chickens at frequencies similar to their corresponding inocula (Table 4 and Supplementary Table 2). Interestingly, two chickens out of five from the rvv-p12/7DMA/BF group presented both Ala270Glu and Ala270Thr mutations (Table 4). No dominant mutation was detected in rvv-p0/BF.

In this segment, the values for the mean number of SNVs, the mean numbers of NS- and S-SNVs, the mutation frequency as well as the nucleotide diversity (Table 4) were always higher for rvv-p12/BF and rvv-p12/7DMA/BF than for rvv-p0/BF. These parameters were similar between rvv-p12/BF and rvv-p12/7DMA/BF, with the exception for the mean number of NS-SNVs, which was 2 times higher in rvv-p12/BF than in rvv-p12/7DMA/BF.

#### Analysis of Segment B From rvv-p0/BF, rvv-p12/BF, and rvv-p12/7DMA/BF

No mutation was found in rvv-p0 (Supplementary Table 4), while three new SNVs were detected in rvv-p0/BF. Two mutations were located in the 5′UTR (in one nucleotide position) and one in VP1 ORF (Table 4). The latter reached a mutation frequency of 2.5 sub/10^4^ nt.

New SNVs were detected in rvv-p12/BF with respect to rvv-p12, distributing in two nucleotide positions in the 5′UTR and three in VP1 ORF (Table 4). VP1 ORF mutation frequency was similar to that found in rvv-p0/BF.

The new SNVs found in rvv-p12/7DMA/BF with respect to rvv-p12/7DMA were distributed in one nucleotide position in the 5′UTR and three in VP1 ORF (Table 4). The mutation frequency in VP1 ORF increased 4.5 and 3 times with respect to those detected in rvv-p0/BF and rvv-p12/BF, respectively.

Concerning dominant mutations, one dominant mutation (Phe138Phe) was found in rvv-p0/BF, although only in one chicken: this variant produced a synonymous mutation (Table 4). One dominant mutation was detected in rvv-p12/BF in the 3′UTR, but its frequency could not be quantified in rvv-p12 inoculum after DNAseq, due to the partial amplification strategy used for this inoculum. However, this mutation was detected in the three replicates when rvv was passaged 12 passages in chicken B cells (Table 1), excluding its appearance after the infection of chickens. Four out four chickens in the rvv-p12/7DMA/BF presented the Thr329Ala mutation in VP1 ORF with variant frequencies similar to those found in the rvv-p12/7DMA inoculum (Table 4 and Supplementary Table 2).

Although infection in chickens was carried out in the absence of 7DMA, rvv-p12/7DMA/BF showed the highest nucleotide diversity and mutation frequency in segment B without reversion in VP1 at position 329 (Table 4). The latter suggests that this mutation shows a relative stability and may contribute to the reduced pathogenicity observed in chickens infected with rvv-p12/7DMA.

### Analysis of Naturally Occurring IBDV Genomes for the Occurrence of Mutations Obtained After Passaging

To investigate if the mutations found in the present study after passages in chicken B cells, DF-1 cells and chickens could be present in other IBDV strains, full length sequences of segments A and B of IBDV were downloaded from Genbank database. A maximum of 192, 123 and 152 sequences were analyzed, corresponding to VP5, polyprotein and VP1 sequences, respectively (Table 5).

**TABLE 5:** Occurrence of mutations observed following experimental passages in naturally occurring IBDV genomes.

IBDV segment	Viral protein	aasubstitution^a^	Virus stock	number ofsamples where the substitution was found/total	Other IBDVstrains with similar residues^b^
A	VP5	Ser7Gly	rvv-p12	1/3	Faragher 52/70 (cv, HG974565.1); USC2003 (vv, KU183665.1); USC2010 (vv, KU183667.1)
	VP5	His63Tyr	rCu-1-p12/DF-1/7DMA	1/3	23/82 (serotype II, Z21971.1)
	VP5	Ile74Phe	rCu-1-p12/B	1/3	Phenylalanine conserved in vvIBDV strains
	VP5	Thr131Ala	rCu-1-p12/DF-1	1/3	GX-NNZ-11 (vv, JX134483.1)
	VP5	Arg133Trp	rCu-1-p12/B/7DMA	1/3	Tryptophane conserved in vvIBDV strains
	VP2	*Pro222Leu	rCu-1-p12/B	1/3	160019 (reassortant isolate, KY610529.1)
	VP2	Gln249Arg	rCu-1-p12/DF-1	1/3	IC-IBDV-Br (KC603937.1); P2 (cv, X84034.1); Gt (cv, DQ403248.1)
	VP2	*Ala270Glu	rvv-p12	3/3	UPM94/273 (vv, AF527039.1); OH (serotype II, U30818.1)
	VP2	*Ala270Thr	rvv-p12/7DMA	3/3	Threonine highly conserved in cvIBDV strains
	VP4	*Cys680Tyr	rCu-1-p12/B, rCu-1-p12/B/7DMA, rCu-1-p12/DF-1, rCu-1-p12/DF-1/7DMA	8/12	Tyrosine highly conserved in vvIBDV strains
	VP4	Cys680Arg	rCu-1-p12/B	1/3	23/82 (serotype II, Z21971.1)
	VP4	*Ala713Thr	rCu-1-p5/ch, rCu-1-p12/B, rCu-1-p12/B/7DMA	4/7	Threonine presents in all sequences analyzed
	VP4	Tyr741His	rvv-p12, rvv-p12/7DMA	4/6	100056 (reassortant isolate, KU234528.1)
B	VP1	*Thr329Ala	rvv-p12/7DMA, rCu-1-p12/B/7DMA, rCu-1-p12/DF-1/7DMA	9/9	IBD13HeB01 (reassortant isolate, AKD94180.1)
	VP1	Val865Ala	rCu-1-p12/B/7DMA, rCu-1-p12/DF-1/7DMA	2/6	HBDY-1 (KX592159.1); OH (serotype II, U30819.1)

^a^Amino acid (aa) substitution indicated using numbering of VP5 ORF, polyprotein or VP1 ORF. ^b^The IBDV classification belonging to serotype I [classical virulent (cv); very virulent (vv)], serotype II or defined as a reassortant isolate was indicated when it was possible. In brackets, Genbank accession numbers. *Dominant mutations.

#### In Segment A, Regardless of 7DMA Treatment

In rvv, 4 different NS-SNVs detected at p12 were present in other IBDV strains, belonging to classical virulent, very virulent strains, reassortant isolates and strains of serotype II (Table 5). Among these four, only 2 were dominant: Ala270Glu and Ala270Thr.

In rCu-1, 6 and 4 several NS-SNVs detected at p12 in chicken B cells and DF-1 cells, respectively, and one NS-SNV detected at p5 in chickens were present and observed in various strains as well as in rvv. Two mutations detected in VP5 (Ile74Phe and Arg133Trp) as well as the dominant mutation in VP4 (Cys680Tyr) appeared very conserved in very virulent strains. The acquisition of those residues, present in vvIBDV strains, could thus be beneficial during rCu-1 passages.

#### In Segment B

Only 2 different SN-SNVs detected under 7DMA treatment in rvv and rCu-1 were present in naturally occurring IBDV genomes. The acquisition of alanine at position 329 in VP1 ORF (dominant mutation Thr329Ala), was detected in a single sequence belonging to a reassortant isolate (IBD13HeB01).

## Discussion

The present study investigates the evolution by serial passaging of two genetically homogeneous virus stocks generated by reverse genetics from infectious clones representing two IBDV viral strains (an attenuated classical virulent, rCu-1, in chicken B cells and DF-1 cells and a very virulent strain, rvv, in only chicken B cells) with and without a selection pressure imposed using the antiviral bases analog 7DMA. It also investigates the pathogenicity of passaged rvv in chickens.

### A Higher Mutation Frequency and Nucleotide Diversity Was Generated in Segment A Than in Segment B, Regardless 7DMA Treatment and Viral Strain

The increase in mutation frequency and genetic diversity can help viruses to adapt to new environments, but this needs to be in compromise with genetic information conservation. In this concern, an important remark to maintain IBDV genetic integrity is the upper limit in mutation frequency that may be tolerated by IBDV. In the present study, the highest mutation frequency value was observed in segment A from rvv-p12/BF (27.6 mutations/10^4^ nt), mainly due to mutations occurring in VP2 and VP3 CDRs. In segment B, the highest values were always reached under 7DMA treatment, with an upper limit on mutation frequency in the range of 11.8–15.9 mutations/10^4^ nt. Similar values to that one found in segment B were observed in CVB3 low fidelity variants (Gnadig et al., 2012), which were considered to be very high mutation frequencies. The increase in viral diversity, determined through nucleotide diversity (π), from p0 to p12 in cells or p0 to p5 in chickens in both segments and in both viruses (with exception for rCu-1-p12/DF-1 in segment B) was expected, since genetically homogeneous virus stocks generated by reverse genetics were used in p0. A similar expansion in viral diversity was previously observed in a Coxsackie virus B3 (CVB3) clone recovered from an infectious cDNA plasmid and serially passaged in two immortalized cell lines (Borderia et al., 2015). Again, segment A showed the most genetic diversity, where the highest values were reached by rCu-1-p12/B and rCu-1-p12/B/7DMA. Why these values were higher in chicken B cells than in DF-1 cells could be explained by a stronger bottleneck effect, driven maybe by a lower MOI used in chicken B cells than in DF-1 cells. Recently, a study on rhesus rotavirus populations indicated a positive correlation between the increase of the genetic diversity and a lower MOI (Kadoya et al., 2020). However, it happened only for segment A, while the values for segment B were similar regardless the presence of 7DMA, the virus strain or the biological system used, suggesting a lower genetic plasticity of segment B. This fact could indicate that, at least in segment A, rCu-1 evolution is cell type-dependent. Therefore, the maximum complexity of a mutant spectrum was reached in segment A, especially when rCu-1 was passaged.

A possible explanation for the high values in genetic diversity and mutant frequency in segment A and for rCu-1 could be the increase in rCu-1 replicative fitness in comparison with rvv, which is characterized by its already high fitness, as documented by high viral loads in the BF upon *in vivo* infection (Le Nouen et al., 2012). The Ile74Phe, Thr131Ala, Arg133Trp mutations in VP5, Cys680Tyr mutation in VP4 and U3205C change in 3′UTR, could support this assumption since the residues Phe74, Ala131 and Trp133 in VP5, Tyr680 in VP4 and C3205 in the 3′UTR are present in vvIBDV strains (this assumption is further discussed in section 4.3).

These results show, for the first time, the increase in IBDV genetic diversity in response to selection pressures.

### Chicken B Cells Did Not Select Known VP2 Mutations Associated to Cell Culture Adaptation in rvv

Many cell culture systems have been used in the study of IBDV. Most of these systems carry the inconvenience that they require modifications in hVP2, especially for non-cell culture adapted viruses, resulting in their attenuation *in vivo*. The rescue of rvv-p0 was recently carried out successfully in primary chicken B cells without modification in hVP2 (which spans from positions 206 to 350) (Cubas-Gaona et al., 2020) as evaluated by Sanger sequencing and confirmed in the present study by NGS.

**FIGURE 5:**
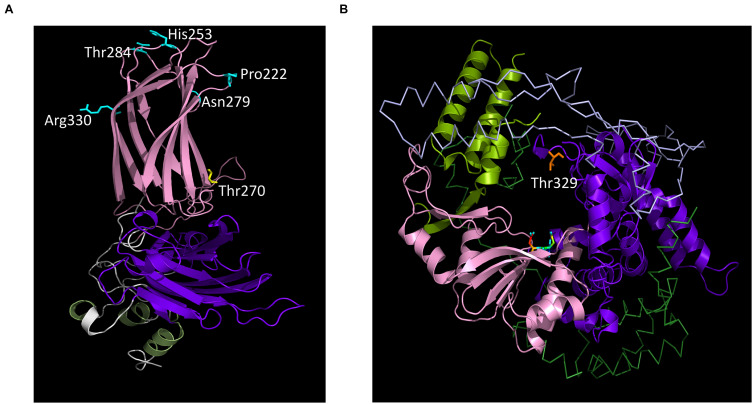
IBDV capsid protein and polymerase structures. **(A)** Ribbon diagram of VP2. The known structure of capsid protein for the cell culture adapted Ct strain (Coulibaly et al., 2005) is shown. The base, shell and projection domains are colored in green, purple, and pink, respectively. Cyan sticks shown residues identified in neutralization escape mutants (position 222) and cell culture adaptation (positions 253, 279, 284, and 330). Position 270 is shown in yellow. **(B)** Ribbon diagram of VP1. The known structure of IBDV polymerase (Pan et al., 2007) is shown. The fingers, palm, and thumb domains are shown in purple, pink, and green, respectively. The active site located from position 401 to 403 is shown in rainbow color and position 329 in orange. The N- and C-terminal domains are shown in line and colored in clear blue and dark green, respectively.

Here, 12 passages of rvv-p0 in chicken B cells did not lead, in spite of an increased genetic diversity compared to rvv-p0, to the apparition of mutations in VP2 positions 253, 279, 284 or 330 previously described as required for *in vitro* cell culture adaptation and/or altering the virulence *in vivo*. However, VP2 Ala270Glu or Ala270Thr mutations, which are also located into hVP2, were detected as dominant mutations in rvv-p12 and rvv-p12/7DMA, respectively. Unlike positions 253, 279, 284 or 330, VP2 position 270 is located at the base of the projection domain (P) (Figure 5A), one of the three domains present in VP2 (Coulibaly et al., 2005). The VP2 Ala270Glu mutation has been previously observed in two vvIBDV strains (OKYM and DV86) after three passages in DT40 cells, an avian leukosis virus-induced chicken B cell line (Terasaki et al., 2008). In addition, glutamic acid in position 270 of VP2 has been reported in another vvIBDV strain which produced low mortality rate (Hoque et al., 2001), suggesting a possible role of this amino acid in the pathogenesis in chickens. Therefore, this mutation may contribute to the reduction in mortality (although not statistically significant in the present study) in chickens infected with rvv-p12. Ala222Val, another substitution in hVP2, was present in the three replicates in rvv-p12, at low variant frequencies (10, 11 and 13%) and persisted after infection *in vivo*. Although position 222 is well exposed at the top of VP2, no available information exists about its role in cell culture adaptation and virulence changes, but this position has been identified as contributing to IBDV antigenicity (Schnitzler et al., 1993; Eterradossi et al., 1997; Samy et al., 2020). Further studies are needed to confirm the role of mutations at VP2 positions 222 and 270. In particular, it is unclear if one or both the dominant mutations detected in rvv-p12, namely VP2 Ala270Thr and/or the mutation in segment B 3′UTR, have an impact on viral phenotype.

### rCu-1 Passage in B Cells Partially Mirrors *in vivo* Evolution

Two mutations were detected in rCu-1-p0 at low frequencies (below 10%). These mutations were not found after 12 passages in chicken B cells nor after 5 passages in chickens, which indicates that these mutations were transient. Since the rCu-1-p0 sequence used in the present study was based on a cell culture adapted Cu-1 strain (Nick et al., 1976), reversion from histidine to glutamine (wild-type residue) at position 253 was expected after passages. Mutations in VP2 at position 253, such as, in most cases, His253Gln, but also His253Asn or His253Asp were indeed observed in vaccine-related viruses isolated from chickens in the field (Olesen et al., 2018). However, in the present study, passages in chickens promoted the apparition of a leucine instead of a glutamine at position 253 in 100% of the viral population, while histidine 253 remained stable in chicken B cells and DF-1 cells. Analysis of complete IBDV genomes available in Genbank revealed that this 253Leu residue was not previously reported. The impact of this residue on viral properties deserves further studies. The lack of mutation of rCu-1 in VP2 position 253 after passages in B cells indicates that additional constraints, present *in vivo*, are required to select changes in this position. In this aspect, chicken B cells did not mirror IBDV evolution *in vivo*.

Surprisingly, the study of IBDV sequences from Genbank revealed the strict conservation of a threonine in position 713 in VP4. The presence of an Alanine at position 713 thus appears as a uniqueness of the rCu-1 sequence used in the present study. It nevertheless illustrates how interesting is the use of chicken B cells, since reversion to a threonine was observed in both those cells and in chickens, but not in DF-1 cells.

Although the Cys680Tyr mutation was only found in chicken B cells and DF-1 cells passaged rCu-1, this position has acquired relevance by belonging, together with four others residues to a VP4 genetic signature of virulence. Recently, Dulwich et al. (2020) demonstrated that VP4 antagonizes type I IFN induction in a strain-dependent manner. Specifically, vvIBDV strains, which harbor a tyrosine in this position, possess an enhanced ability in IFN antagonism compared with classical viruses, which have a cysteine in this position. It is thus tempting to speculate that the acquisition of this change in rCu-1 could promote higher viral fitness, by better inhibiting innate immunity. However, why this was not observed for rCu-1 passaged in chickens remains an open question. During *in vivo* passaging, in spite of the nearly 100 fold increase of the mean viral titer in the bursae, chickens did not develop any clinical sign, which indicates that mutations that arose during the *in vivo* passages are not sufficient to render the virus pathogenic. Further work, using reverse genetics, is necessary to dissect which mutations contribute to this increased replicative fitness *in vivo* and whether this goes with microscopic lesions of the bursa or immunosuppression.

Finally, the U3205C change, located in the 3′UTR sequence of segment A, found exclusively in rCu-1-p12/B/7DMA, is highly conserved in vvIBDV strains (Xia et al., 2008). This mutation could provoke changes in the RNA secondary structure and enhance the virulence as previously suggested (Xia et al., 2008).

### VP1 Thr329Ala in IBDV Viral Polymerase Together With VP2 Ala270Thr Could Be Responsible for the Reduced Pathogenicity Provoked by rvv-p12/7DMA

Alteration of viral polymerase fidelity has allowed a better understanding of viral evolution and adaptation (Pfeiffer and Kirkegaard, 2003; Coffey and Vignuzzi, 2010; Gnadig et al., 2012). Nucleoside analogs, such as ribavirin and 5-fluorouracil, have been classically used as a selective pressure in order to select viral resistant mutants (Borderia et al., 2016). The inhibitory effect of 7DMA on IBDV replication was previously addressed (Cubas-Gaona et al., 2018). In the present study, the detection in p12 of a unique non-synonymous and dominant mutation, VP1 Thr329Ala in segment B of both rvv and rCu-1 and in both chicken B cells and DF-1 cells, suggested that a resistance mechanism was established throughout the passages to overcome the mutagenic effects of 7DMA. Deep-sequencing of the intermediate viral stocks would be necessary to thoroughly address this point; this approach is beyond the scope of this study whose objective was to identify relevant mutations in the final viral stocks.

The alignment results indicated that Thr329 is present in 147 out of 152 published segment B sequences in NCBI database, which suggests this residue is highly conserved and important during viral genome replication and transcription. IBDV polymerase contains fingers, palm and thumb subdomains and, as most polymerases, has the shape of a right hand (Pan et al., 2007). Thr329 is located inside the finger subdomain in a 15-residue loop (residues 321–335) forming a fingertip structure (Pan et al., 2007), which is a unique feature of RdRps. This structure helps to form template-binding and NTP channels through which NTPs would reach the active site (Choi, 2012). In Figure 5B, residue Thr329 is shown (in orange), which appears distant from the active site residues (in rainbow color). Similar locations for mutations, in finger subdomain, were detected in other viral high-fidelity, nucleoside analog ribavirin-resistant polymerases, such as Gly64 in poliovirus (Pfeiffer and Kirkegaard, 2003) and Leu123 in Human enterovirus 71 (Meng and Kwang, 2014).

During chicken infection, carried out in absence of 7DMA, rvv-p12/7DMA/BF viral population showed to be the most genetically heterogeneous in segment B, with a stable Thr329Ala mutation. Therefore, the *in vivo* attenuation of rvv-p12/7DMA, shown by the significant reduction in mortality rate in spite of otherwise unchanged phenotypic traits (such as viral titer in BF, b/B ratio, bursal atrophy and blood B cells depletion), could be the result of its more diverse genetic population harboring an increased percentage of genomes with reduced viral fitness. This fact could compromise viral replication and dissemination, and together with Ala270Thr mutation in VP2 contribute to the observed *in vivo* attenuation. This population with lower fitness could be the main reason whereby viral shedding in the rvv-p12/7DMA group was lower at 3 and 4 d.p.i. compared with that of the other rvv groups. Similarly, the statistically significant splenomegaly at 4 d.p.i. in the rvv-p12/7DMA group may be due to this high genetic diversity and/or to a reduced replicative fitness leading to a higher activation of the inflammatory response.

In conclusion, this work, through the use of genetically homogeneous virus stocks, illustrates the evolution of IBDV in various host systems. In these conditions, segment A showed the highest genetic variation. The various dominant changes identified in this study such as His253Leu, Ala270Glu and Ala270Thr in VP2, Cys680Tyr in VP4 and Thr329Ala in VP1 represent candidate virulence markers and merit further investigation using IBDV reverse genetics.

This study illustrates also how NGS sheds new light on the existence of minority mutants, which may have been unnoticed in previous studies relying on Sanger sequencing; these mutations may modulate viral proteins properties and may contribute to shape viral phenotypes.

## Data Availability Statement

The data presented in the study are deposited in the BioSample database at NCBI under accession numbers from SAMN14642839 to SAMN14642909. The data is publicly available.

## Ethics Statement

All animal trials were conducted in an animal facility approved for animal experiments (n° C-22–745–1) and were approved by ANSES Ploufragan local committee for animal welfare; chickens were raised and humanely euthanized in agreement with EU directive number 2010/63/UE. Serial passages of rCu-1-p0 in chickens were approved by ANSES Ethical Committee, registered at the national level under number C2EA-016/ComEth ANSES/ENVA/UPEC and authorized by French Ministry for higher education and research under permit number APAFiS#2494-201512021054584v1. Pathogenicity assessment in SPF chickens was approved by ANSES Ethical Committee, registered at the national level under number C2EA-016/ComEth ANSES/ENVA/UPEC and authorized by French Ministry for higher education and research under permit number APAFiS#4945-20 16041316546318 v6.

## Author Contributions

LLC-G carried out experiments, analyzed the results, and wrote the manuscript. AF analyzed the data from NGS. LLC-G, CC, and SS carried out the animal experiments. CC analyzed blood samples by flow cytometry. F-XB carried out the analysis of mutations present IBDV genomes from databases. MC modeled the non-coding regions. SB gave support in the statistical analysis. YB, PL, HQ, and AL carried out the NGS. PL carried out BioSample submission. MA supervised technically the animal experiment. NE, BG, PAB, and AK revised the manuscript. SS conceived the study, supervised LLC-G and CC and revised the manuscript. All authors contributed to the article and approved the submitted version.

## Conflict of Interest

The authors declare that the research was conducted in the absence of any commercial or financial relationships that could be construed as a potential conflict of interest.

## Footnotes

1 https://www.ncbi.nlm.nih.gov/genbank/

## Supplementary Material

Supplementary Figure 1 Gating strategy used for the identification of thrombocytes (CD45^+^ K1^+^), monocytes (CD45^+^ K1^+^Kul01^+^), chicken B cells (CD45^+^ Bu1^+^), and T-cells (CD45^+^ CD4^+^, CD45^+^CD8^+^ and CD45 TCRγδ^+^) based on marker expression followed by FSC/SSC characteristics.

Supplementary Figure 2 Blood counts of T cells, thrombocytes and monocytes in chickens infected with rvv-p0, rvv-p12, or rvv-p12/7DMA. Cell concentrations in blood at 2 and 21 days post-inoculation (d.p.i.) (*n* = 10 chickens per group except for the rvv-p0 group). Points represent individual values; boxplots indicate median and interquartile range. For all graphs, different letters indicate a *p*-value < 0.05 between the different groups using Kruskal-Wallis test followed by Fisher’s least significant difference test with Holm adjustment method for multiple comparisons. NI, non-infected group.
