# Supplementary figures and images for: Genome Evolution of Two Genetically Homogeneous Infectious Bursal Disease Virus Strains During Passages in vitro and ex vivo in the Presence of a Mutagenic Nucleoside Analog
Source: Front Microbiol. 2021 Jun 11;12:678563. doi: 10.3389/fmicb.2021.678563 (PMC8226269; doi:10.3389/fmicb.2021.678563)

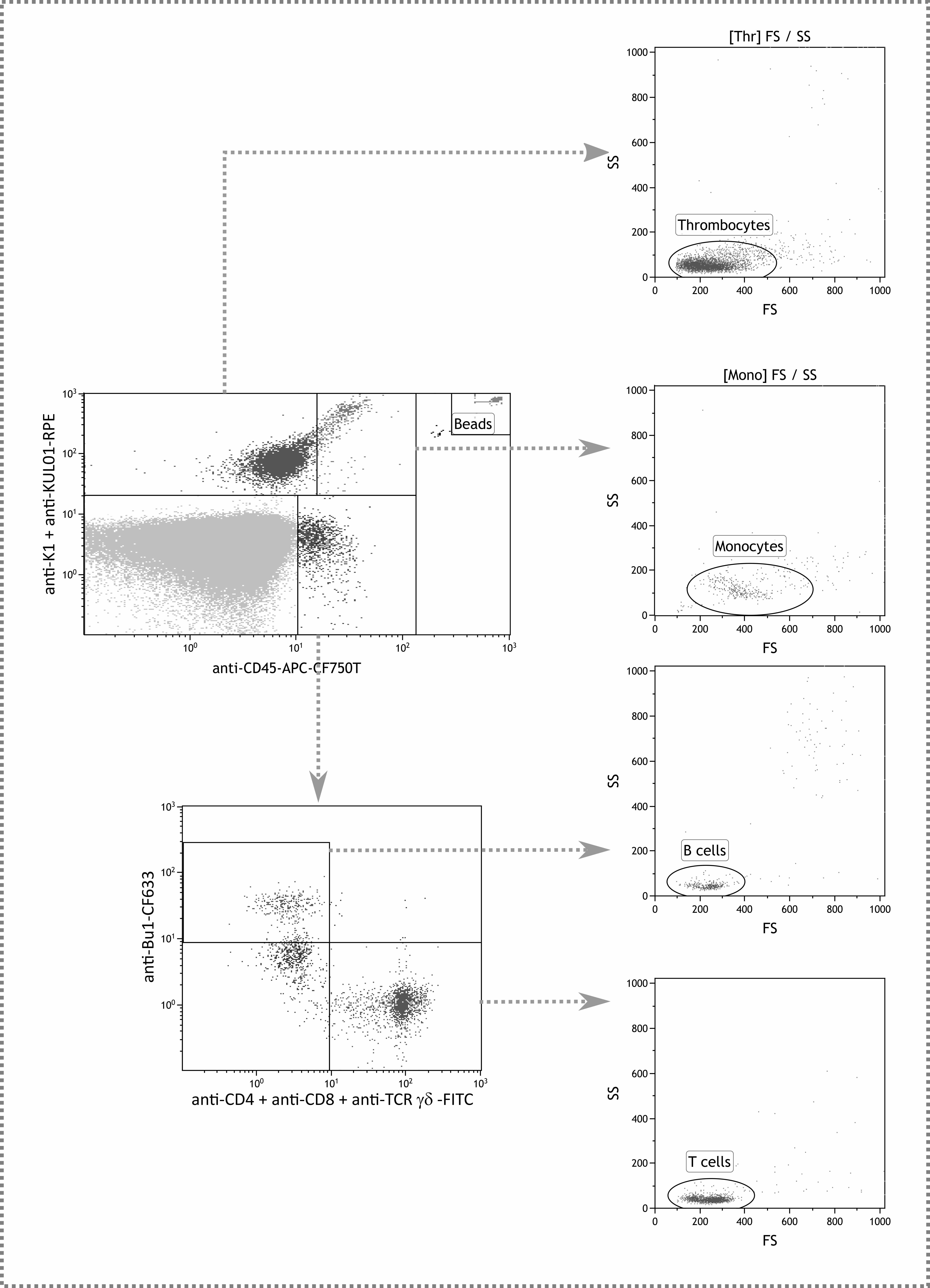

Supplement: Supplementary Figure 1 — Gating strategy used for the identification of thrombocytes (CD45+ K1+), monocytes (CD45+ K1+Kul01+), chicken B cells (CD45+ Bu1+), and T-cells (CD45+ CD4+, CD45+CD8+ and CD45 TCRγδ+) based on marker expression followed by FSC/SSC characteristics. [file Image_1.JPEG]

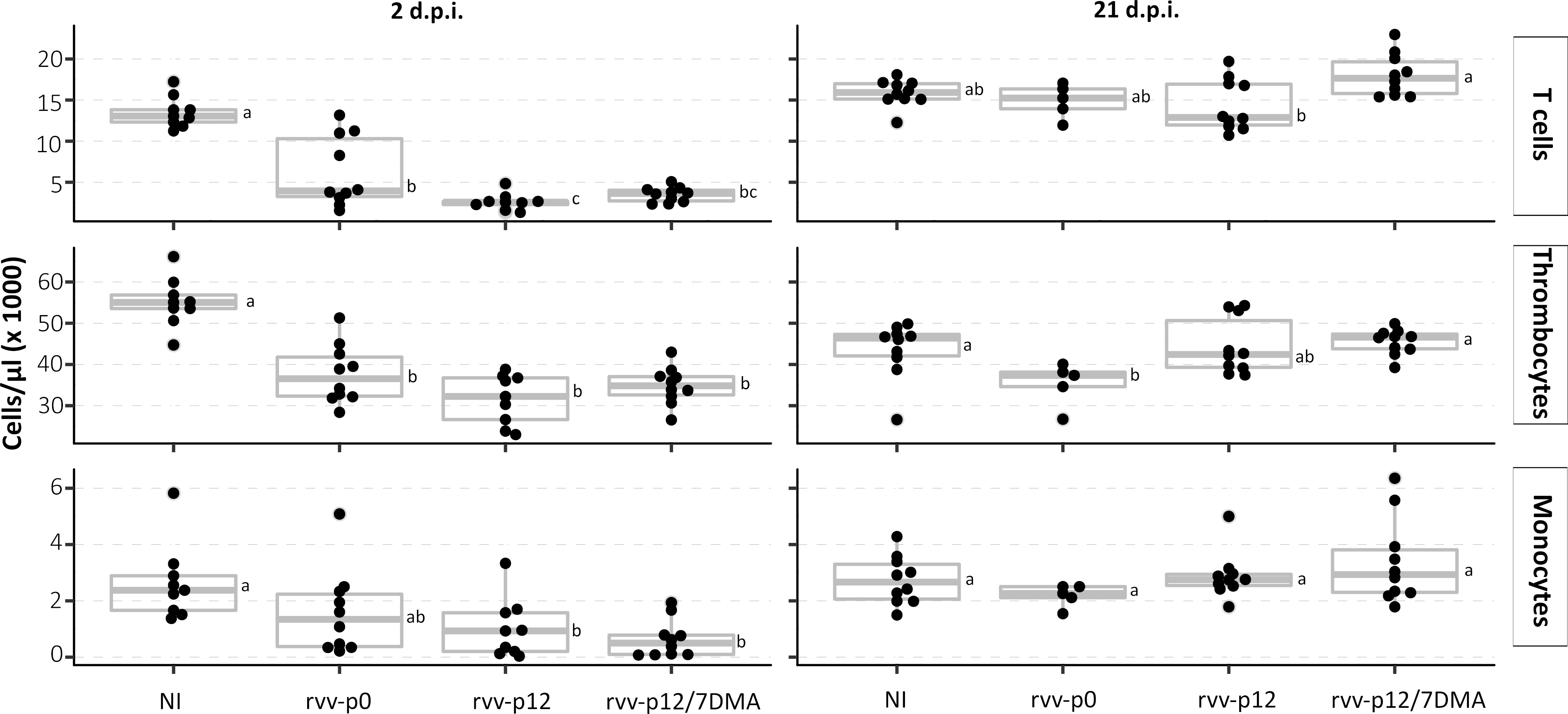

Supplement: Supplementary Figure 2 — Blood counts of T cells, thrombocytes and monocytes in chickens infected with rvv-p0, rvv-p12, or rvv-p12/7DMA. Cell concentrations in blood at 2 and 21 days post-inoculation (d.p.i.) (n = 10 chickens per group except for the rvv-p0 group). Points represent individual values; boxplots indicate median and interquartile range. For all graphs, different letters indicate a p-value < 0.05 between the different groups using Kruskal-Wallis test followed by Fisher’s least significant difference test with Holm adjustment method for multiple comparisons. NI, non-infected group. [file Image_2.JPEG]
